# Minor and inconsistent differences in Big Five personality traits between vegetarians and vegans

**DOI:** 10.1371/journal.pone.0268896

**Published:** 2022-06-08

**Authors:** Markus Müssig, Tamara M. Pfeiler, Boris Egloff

**Affiliations:** 1 Johannes Gutenberg University Mainz, Mainz, Germany; 2 Leibniz Institute for Resilience Research Mainz, Mainz, Germany; Max Planck Institute for Human Development, GERMANY

## Abstract

Most research examining individuals who follow different diets has combined vegetarians and vegans into a single group. To investigate whether this consolidation is justified, we analyzed possible differences between vegetarians and vegans for the Big Five personality traits in two studies. In our pre-study, we used data from a German convenience sample of 400 vegetarians and 749 vegans and found that vegans reported slightly higher scores in Openness compared to vegetarians (*d* = 0.22). In the preregistered main study, we used data provided by 1203 vegetarians and 128 vegans from the German Socio-Economic Panel Study; we found that vegetarians reported slightly higher scores in Neuroticism compared to vegans (*d* = 0.18) but did not differ in Openness. We found no differences in Conscientiousness, Extraversion, or Agreeableness in either study. Controlling for the socio-demographic variables of age, gender, and socio-economic status did not alter the pattern of results. Overall, these results suggest that there are no or only small differences in Openness or Neuroticism between vegetarians and vegans. Further studies utilizing very large, representative samples are needed to better understand the relationship between personality and diet groups.

## Introduction

According to recent polls, around 8% percent of people in Western countries follow a vegetarian or vegan diet [1]. In accordance with these figures, a recent Appinio poll [2] found that 6% of German adults followed a vegetarian diet and around 2% followed a vegan diet. In the US, a Gallup poll [3] found that 5% of adults followed a vegetarian diet and 3% followed a vegan diet. Since this equates to several million people, these dietary groups have been of increasing interest to research [4, 5]. The characteristics of vegetarians have been studied for quite some time, with most studies focusing on differences between vegetarians and omnivores [6–8]. Vegans, on the other hand, are rarely investigated as a separate group [9, 10], and are often combined into a single group with vegetarians [6, 7, 11–15]. Here we explore whether this consolidation is justified, or whether vegetarians and vegans should be differentiated with regard to personality.

### Why it might be useful to differentiate between dietary groups

*Vegetarians* are most commonly defined as people who exclude meat from their diet but who consume other animal products such as milk, eggs, or leather. *Vegans* are defined as people who exclude all animal products from their consumption. While these definitions are subject to debate [16], they are a useful basis on which to investigate differences between groups. Given these definitions, there are at least three distinct categories of consumers regarding animal products: omnivores (not excluding any specific product), vegetarians (excluding some animal products), and vegans (excluding all animal products). Since all three categories likely reflect different attitudes toward food and animals, as well as related life-styles, it seems reasonable to assume that there are also systematic differences between the people who belong to each category. From the perspective of psychological research, a powerful and well-established framework to investigate such inter-individual differences at the highest hierarchical level is the Big Five personality traits.

The Big Five traits encompass *Openness* (e.g., creativity, intellect), *Conscientiousness* (e.g., order, reliability), *Extraversion* (e.g., sociability, energy), *Agreeableness* (e.g., helpfulness, trustfulness), and *Neuroticism* (e.g., irritability, vulnerability) [17]. Since these traits are broadly defined, they capture a large variety of information. Accordingly, several reviews of the literature have shown that the Big Five personality traits predict behaviors and important life outcomes above and beyond socioeconomic status or cognitive ability [18, 19]. Conscientiousness and Extraversion, for example, have been linked to a decreased risk of mortality, even when controlling for factors such as education or age. Neuroticism has been positively associated with the probability of divorce [19], Agreeableness has been negatively associated with the risk of heart disease [20], Conscientiousness has been negatively associated with risk-taking behavior [20], and Openness has been shown to predict both substance abuse and the trajectory of acculturation processes [20].

Considering the important relationships between the Big Five personality dimensions and many life outcomes, exploring these traits might further improve our understanding of how and why there are inter-individual differences between people in different dietary groups, and might highlight the need to differentiate between those dietary groups at a more granular level. Consider a study that links a vegetarian or vegan diet to a decreased mortality or cancer rate compared to an omnivore diet. By not considering that there might be systematic differences in personality between these dietary groups that go beyond their diet, the reported effect might be an over- or underestimation of the true effect. However, despite this important consideration, to our knowledge, research into personality differences between different dietary groups is scarce.

### What do we know about Big Five differences between dietary groups?

Almost all studies investigating the consumption of animal products and the Big Five have focused on differences between vegetarians (often including vegans, see [21], for an overview) and omnivores, or on the association between the Big Five and the amount of meat consumed by omnivores. Since vegans are rarely investigated as a standalone group, we focus on research comparing vegetarians and omnivores first and then attempt to apply these results to the vegetarian vs. vegan comparison.

These studies showed inconsistent results: Keller and Siegrist [22] analyzed a random Swiss sample from a telephone survey (*N* = 951) and found that Openness and Agreeableness were positively associated with meat consumption, while Extraversion was negatively associated with meat consumption. In contrast, in a large Estonian sample (*N* = 1,691), Mõttus, Realo [23] found that Openness was negatively associated with meat consumption. A study by Pfeiler and Egloff [24] utilizing two large samples (each *N* > 4,000) representative for the German population found that Openness and Agreeableness were negatively associated with meat consumption. In a later study using a representative sample of 13,892 Australians (taken from an Australian household panel study), Pfeiler and Egloff [25] found Openness to be negatively associated with meat consumption while Extraversion and Neuroticism were positively associated with meat consumption.

While associations between personality and the amount of meat eaten are informative and important to consider, they do not allow researchers to explore differences between different dietary groups such as omnivores and vegetarians, or vegetarians and vegans. Only three studies to date have investigated systematic differences between vegetarians and omnivores with regard to the Big Five. In a sample of psychology students, including 276 veg*ns (a group combining vegetarians and vegans) and 4955 omnivores, Forestell and Nezlek [14] found that veg*ns were higher in Openness and Neuroticism compared to omnivores. Tan, Conner [11] investigated Big Five differences between veg*ns and omnivores in three different samples. Each sample included 30 to 100 veg*ns and several hundred omnivores. In one sample, they found that veg*ns were higher in Openness, while in another sample this group was higher in Agreeableness and Conscientiousness. Overall, no consistent results were found. In the only study that specifically investigated Big Five differences between veg*ns and omnivores in a large representative sample, Pfeiler and Egloff [6] analyzed data from two consecutive years (2014 and 2015) of a subsample of a German household panel study. Data from the first year included 123 veg*ns and 4,373 omnivores, and data from the second year included 306 veg*ns and 4,819 omnivores. The researchers consistently found that veg*ns were higher in Openness, while the first study also pointed toward Conscientiousness being lower in veg*ns. Taken together, the most consistent result seems to be that vegetarians are higher in Openness compared to omnivores.

### Comparing vegetarians and vegans

The aim of this article is to explore possible differences in broad personality traits between vegetarians and vegans. While many prior studies have combined vegetarians and vegans into a single category, there have also been studies that investigated them separately, and which noted systematic differences in different psychological variables between vegetarians and vegans. For example, vegans see their dietary patterns as more of a part of their identity [12, 26], hold a stronger animal rights position [27], and may be seen as less masculine [28]. However, only two studies have investigated Big Five differences between vegetarians and vegans: Kováč and Halama [29] analyzed a small sample of 56 vegetarians and 57 vegans and found no difference in the Big Five between both groups. Kessler, Holler (30) compared a large convenience sample of 4,427 vegetarians and 4,822 vegans and found that vegetarians were slightly higher in Neuroticism (*d* = 0.14) and slightly lower in Openness (*d* = 0.14) than vegans. While the study by Kessler, Holler [30] was highly powered and analyses were controlled for confounding variables such as age, gender, and education, these results have not been replicated since. Furthermore, no study is available that investigates differences in the Big Five personality dimensions between vegetarians and vegans in a representative sample.

### The present studies

In this article, we used a two-step approach to examine possible differences between vegetarians and vegans in the Big Five personality traits. In a pre-study, we analyzed existing data from a different project, which includes a large convenience sample of vegetarians and vegans to see whether the results by Kessler, Holler [30] are replicable in a similar study design. Informed by the results of the pre-study, we then preregistered our main study (osf.io/5m4hd), which used data from the German Socio-Economic Panel Study, a large sample representative of the German population to see whether these results are robust.

## Pre-Study

### Method

#### Sample

The pre-study sample was recruited via an online questionnaire posted in several food-related social media groups, a university mailing-list, and we had our survey included in the newsletter of one of the largest German non-profit vegetarian associations, *ProVeg International*. *ProVeg International* did not commission the survey and we did not receive financial compensation. The pre-study data was part of a larger project about omnivore, vegetarian, and vegan diets that, altogether, comprised three studies. The Big Five personality traits were assessed in all three studies. For a complete list of all measured variables, please see the project page on Open Science Framework at https://osf.io/bj4gv/?view_only=ea5c58e3b1c1488e924c03a9dc2f9eff.

Participants consented to data collection after being informed about the aims and contents of the survey and were able to withdraw from the survey at any time without penalty. After completion of the online questionnaire, participants could choose between entering a raffle to win a 25-Euro gift certificate (Readers who responded to the survey publicized in ProVeg were entered to win a 50-Euro gift certificate), or to donate the same amount to a non-profit organization. Undergraduate students also received course credit for participating. Data were collected according to ethical standards for the treatment of human subjects. Please note that this type of routine questionnaire study does not require formal approval by the local ethics committee. Data are available at https://osf.io/z4agk/?view_only=b5e0e90628f9480b82f7ecafc4e81518.

In total, 1149 vegetarians and vegans (80.8% women) completed the survey. On average, participants were 38.15 years old (*SD* = 14.77).

### Measures

#### Eating behavior

To assess eating behavior, participants were asked which dietary category they would place themselves into, with choices being ovo-lacto-vegetarian, ovo-vegetarian, lacto-vegetarian, vegan, and ethically motivated vegan. Categories were described as follows: *Ovo-lacto* = includes egg and milk products; *Ovo* = excludes milk but includes egg products; *Lacto* = excludes egg but includes milk products; *Vegan* = excludes animal products from the diet but buys leather or wool products; *Ethically motivated vegan* = does not buy any animal products, including leather, wool, etc. For our analyses, we combined ovo-lacto-vegetarians, ovo-vegetarians, and lacto-vegetarians into the vegetarians group, and vegans and ethically motivated vegans into the vegans group.

#### Socioeconomic status

As a measure of socioeconomic status, we asked participants about their highest completed academic degree and classified them according to the International Standard Classification of Education 2011 [31]. The ISCED-11 ranks levels of education from early childhood education (ISCED level 0) up to a doctoral or equivalent level (ISCED level 8).

#### Big Five

The Big Five personality dimensions were measured with the German version of the BFI-K [32]. The BFI-K is a 21-item questionnaire using a 5-point Likert scale that measures Openness (α = .72) with five items; it also measures Conscientiousness (α = .68), Extraversion (α = .80), Agreeableness (α = .66), and Neuroticism (α = .77) with four items each.

### Results

The pre-study sample consisted of 400 vegetarians and 749 vegans. Vegetarians were 37.11 years old on average (*SD* = 15.21), 320 (80.0%) were women, and they had an average ISCED-11 score of 5.24 (*SD* = 1.71), indicating that most participants in our sample at least finished tertiary education. The vegans were 38.70 years old on average (*SD* = 14.54), 608 (81.1%) were women, and they had an average ISCED-11 score of 5.24 (*SD* = 1.78). Comparisons of sociodemographic variables revealed no difference in socioeconomic status (ISCED-11) or gender distributions between vegetarians and vegans, but vegans were significantly older than vegetarians (*U* = 162,521; *Z* = 2.375; *p* = .018). Our sample allowed us to detect effects larger than *d* = 0.17 with 80% power and *p* = .05.

Regarding the Big Five personality dimensions, we found no differences between vegetarians and vegans in Conscientiousness, Extraversion, Agreeableness, or Neuroticism, but vegans were slightly higher in Openness than vegetarians (*d* = .22, see Table 1). To test whether this difference was caused by the age difference between vegetarians and vegans, we conducted a linear regression analysis and included both age and eating behavior as predictors. When controlling for age, this pattern held, and eating behavior remained a significant predictor for the difference in Openness (β = .104, *p* < .001), again with a small effect size.

**Table 1:** Pre-study: Big five personality traits as a function of eating behavior.

	Vegetarian (*N* = 400)		Vegan (*N* = 749)				
Big Five	*M*	*SD*	*M*	*SD*	*t*	*p*	*d*
Openness	3.96	0.67	4.11	0.65	-3.55	< .001	-0.22
Conscientiousness	3.73	0.66	3.74	0.65	-0.25	0.802	-0.02
Extraversion	3.32	0.84	3.33	0.85	-0.25	0.805	-0.02
Agreeableness	3.20	0.75	3.17	0.77	0.55	0.582	0.03
Neuroticism	3.10	0.82	3.11	0.88	-0.30	0.765	-0.02

Mean values vary between 1 and 5.

### Discussion

Kessler, Holler [30] reported that vegans were higher in Openness and lower in Neuroticism compared to vegetarians. While we found a similar–albeit slightly larger–effect for Openness (*d* = 0.22, compared to *d* = 0.14 found by Kessler, Holler [30]), which held when controlling for age. We found no difference in Neuroticism.

## Main study

The pre-study showed slight deviations from the results of Kessler, Holler [30], namely that we were able to replicate the small difference in Openness, but could not replicate the difference in Neuroticism between vegetarians and vegans. Overall, the difference in openness seems to be the most consistent. We took these results as a starting point for our preregistered main study (preregistration is available from https://osf.io/5m4hd), and explored differences in the Big Five between vegetarians and vegans in a sample representative for the German population, expecting a Big Five difference in Openness only. Since previous studies have shown that there are differences between people in different dietary groups that go beyond the Big Five [6], we took the opportunity to provide exploratory analyses on differences in political interest, health, satisfaction, as well as affective well-being.

### Method

The data used in the main study were provided by the German Socio-Economic Panel (SOEP) of the German Institute for Economic Research [33]. The SOEP-CORE panel is a large longitudinal representative survey of private households and persons in Germany that has been running since 1984. Every year, panel members are interviewed on different economic and political questions; every couple of years, questions on eating behavior (most recently in 2018) and the Big Five personality dimensions (most recently in 2017) are included. Our research did not require ethical approval because we analyzed existing and fully anonymized data; informed consent was obtained from participants by the German Institute for Economic Research. Data are available from the German Institute for Economic Research.

### Sample

In 2018, the SOEP interviewed 30,306 panel members, of which 1,414 (4.67%) reported eating a mostly vegetarian diet and 162 (0.53%) reported eating a mostly vegan diet. Since we were interested in differences in personality, we preregistered that we would only include individuals in our final sample who provided answers on all items of the personality questionnaire in the previous year; this resulted in a sample of 1,203 vegetarians and 128 vegans. These 1,331 vegetarians and vegans were 45.54 years old on average (*SD* = 16.64) and mostly women (74.4%).

### Measures

#### Eating behavior

Eating behavior was assessed with a single item (“Do you follow a mainly vegetarian or vegan diet?”).

#### Socioeconomic status

As a measure of socioeconomic status, the SOEP provides the International Socioeconomic Index of occupational status (ISEI-08). The ISEI was developed by Harry Ganzeboom with the goal of providing a standardized, continuous, and empirically grounded measure of socioeconomic status. This metric is used in large-scale international studies such as the PISA studies (Programme for International Student Assessment) and has since been refined using a larger dataset [34]. The ISEI-08 assumes that the effect of education on income is mediated by a person’s occupation. The ISEI-08 maximizes this indirect effect and therefore provides a more detailed measurement of socioeconomic status than education or income alone in a single value. We preregistered that we planned to control our analyses for education and income as a measure of socioeconomic status. During analysis, we found out that the SOEP provides the ISEI-08 value, which meaningfully combines both variables, and we therefore chose to go forward using this measure.

#### Big Five

The Big Five personality traits were measured using a 16-item German short version of the Big Five Inventory (BFI-S, [35]). The BFI-S is a 16-item questionnaire that uses a 7-point Likert scale to measure Openness (α = .64) with four items, as well as Conscientiousness (α = .64), Extraversion (α = .75), Neuroticism (α = .66), and Agreeableness (α = .47) with three items each. Internal consistencies as an indicator of reliability should be interpreted with caution, as, to maximize validity, the three items were each explicitly selected to represent different aspects of each broad Big Five dimensions. Because of this heterogeneity, it has been suggested that test–retest reliabilities are more adequate measures of reliabilities than internal consistencies in the case of the BFI-S. Indeed, the temporal stability of the BFI-S is substantial, with a mean r_tt_ = .70 at a test–retest interval of 18 months [36].

#### Measures included in exploratory analyses

*Political interest* was assessed with a single item (“How interested are you in politics?”) on a 4-point scale ranging from 1 = not at all to 4 = very strongly. *Current life satisfaction* as well as *satisfaction with health* were assessed with a single item each (“How satisfied are you currently with (1) your life in general (2) your health?”) on a 11-point scale ranging from 0 = very unsatisfied to 10 = very satisfied. *Current health status* was assessed with a single item (“How would you describe your current health status?”) on a 5-point scale ranging from 1 = badly to 5 = very well. *Affective well-being* was assessed with four items that asked about the frequency of experiencing pleasant or unpleasant emotional states (e.g. happiness or anger) on a 5-point scale ranging from 1 = very rarely to 5 = very often.

### Results

The sample in our main study consisted of 1,203 vegetarians and 128 vegans. The vegetarians were 45.69 years old on average (*SD* = 16.61), 909 (75.6%) were women, and they had an average ISEI-08 score (ISEI-08 values were only available for a subset (1,021 vegetarians and 114 vegans) of our sample.) of 54.95 (*SD* = 20.64). The vegans were 44.16 years old on average (*SD* = 16.90), 90 (70.3%) were women, and they had an average ISEI-08 score of 47.50 (*SD* = 22.20). Comparisons of sociodemographic variables revealed no difference in age and gender distributions between vegetarians and vegans, but vegetarians had a significantly higher socioeconomic status than vegans did (*t* = 4.43, *p* = .001, *d* = .036). Our main study sample includes relatively fewer vegans (χ^2^ = 833.11, *p* < .001) and was younger (*p* < .001, *d* = 0.30), compared to the sample used in our pre-study. Our sample size allowed us to detect effects larger than *d* = 0.23 with 80% power and *p* = .05 (one-tailed).

Regarding the Big Five personality dimensions, we found no differences between vegetarians and vegans in Openness, Conscientiousness, Extraversion, or Agreeableness, but vegetarians showed very slightly higher scores in Neuroticism compared to vegans (*d* = 0.18, see Table 2). To test whether this difference is explained by the difference in socioeconomic status between vegetarians and vegans, we conducted a linear regression analysis and included socioeconomic status as well as dietary group as predictors. Since ISEI-08 values were not available for our entire sample, we conducted these robustness checks with a sample of 1,021 vegetarians and 114 vegans. This reduced sample showed no significant differences compared to the original sample regarding age, gender, or socioeconomic status, or regarding the pattern of personality differences found in the original sample (all *p* values > .490, except for Neuroticism which had *p* = .042). When controlling for socioeconomic status, the previously found pattern held and dietary group remained a significant predictor for the difference in Neuroticism (β = -.067, *p* = .033), again with a very small effect size.

**Table 2:** Main study: Big five personality traits as a function of eating behavior.

	Vegetarian (*N* = 1203)		Vegan (*N* = 128)				
Big Five	*M*	*SD*	*M*	*SD*	*t*	*p*	*d*
Openness	5.29	0.98	5.28	1.05	0.10	0.919	0.01
Conscientiousness	5.69	0.94	5.67	0.95	0.29	0.771	0.02
Extraversion	4.82	1.22	4.85	1.15	-0.29	0.772	-0.02
Agreeableness	5.45	0.92	5.43	0.95	0.18	0.856	0.02
Neuroticism	3.89	1.28	3.66	1.14	1.96	0.050	0.18

Mean values may vary between 1 and 7.

### Results from exploratory analyses

Our exploratory analyses showed no differences between vegetarians and vegans in current life satisfaction, satisfaction with health, health status, and affective well-being, but vegetarians were slightly more politically interested compared to vegans (*d* = 0.28, see Table 3). Since vegetarians in our sample had a significantly higher socioeconomic status, and scored slightly higher in Neuroticism, we repeated the exploratory analyses and controlled for both factors by including socioeconomic status (ISEI-08) and Neuroticisms alongside dietary group as predictors in a linear regression model. By controlling for both variables, the differences between vegetarians and vegans in current life satisfaction, satisfaction with health, health status, and affective well-being remained insignificant, but dietary group no longer significantly predicted political interest (β = -.042, *p* = .169).

**Table 3:** Main study: Results of exploratory analyses of political interest, current life satisfaction, satisfaction with health, current health status as well as affective well-being as a function of eating behavior.

	Vegetarian (*N* = 1203)		Vegan (*N* = 128)					
Big Five	*M*	*SD*	*M*	*SD*	*t*	*p*	*d*	*p* ^*a*^
Political Interest	2.54	0.82	2.31	0.78	-3.05	0.002	0.28	0.169
Current Life Satisfaction	7.46	1.73	7.60	1.68	-0.84	0.404	0.08	0.420
Satisfaction with Health	6.83	2.22	7.10	2.24	-1.31	0.191	0.12	0.133
Current Health Status	3.56	1.03	3.65	1.11	0.96	0.336	-0.09	0.261
Affective Well-Being	3.63	0.70	3.72	0.67	-1.38	0.167	0.13	0.408

Mean values of Political interest may vary between 1 (not interested at all) and 4 (very interested). Mean values of Current Life Satisfaction and Satisfaction with Health may vary between 0 (completely unsatisfied) and 10 (completely satisfied). Mean values of Current Health Status may vary between 1 (bad) and 5 (very good) and mean values of Affective Well-Being may vary between 1 (rare experiences of pleasant emotions) and 5 (frequent experiences of pleasant emotions).

^a^ *p*-values of mean differences controlled for socioeconomic status and Neuroticism.

### Discussion

In our main study, we aimed to extend existing research by providing data on personality differences between vegetarians and vegans from a large sample representative for the German population. We expected that vegans would be higher in Openness and that there would be no differences in the other Big Five. Contrary to this assumption, vegans reported slightly lower Neuroticism compared to vegetarians (*d* = 0.18), and we did not find the expected difference in Openness. Additionally, our exploratory analyses showed that, when controlling for the differences in socioeconomic status and neuroticism between vegetarians and vegans, there was no difference in current life satisfaction, satisfaction with health, health status, affective well-being, and political interest between both dietary groups.

## General discussion

In this article, we attempted to contribute to the mapping of differences in personality between vegetarians and vegans. An understanding of the differences in the Big Five between vegetarians and vegans might inform future studies looking into differences in more finely graded psychological variables between both groups, or might be of use to researchers who formulate models of how and why people choose a vegetarian or vegan diet. Furthermore, an improved understanding of these differences might aid research in fields outside of psychology that investigate the effects of diet on health and other important outcomes. The two studies that investigated differences in the Big Five prior to this study either found no difference between both groups [29] or found very small differences (each *d* = 0.14, [30]) in Openness (with vegans reporting higher values) and Neuroticism (with vegetarians reporting higher values). In our preliminary study using a large convenience sample we found slightly higher values in Openness for vegans compared to vegetarians (*d* = 0.22) and no differences in the other Big Five. However, in our main study using a large sample representative for the German population, we only found slightly higher values in Neuroticism for vegetarians compared to vegans (*d* = 0.18).

The different results between our pre-study and our main study might be due to the slightly differing definition of vegetarians and vegans, which might have led to only strict vegetarians or vegans being included in either group in our pre-study (choice between five labeled dietary groups), while the laxer definition in our main study might have blurred the lines between both dietary groups (question about following a *mainly* vegetarian or vegan diet). This line-blurring might have meant that non-strict vegans were in our vegans group, and differences are attenuated as a result (for a similar definition issue see [6]). While we cannot rule out this possibility, we believe that there might be other systematic differences between the convenience samples used by Kessler, Holler [30] as well as in our pre-study, and the representative sample used in our main study, and we suggest future research to rely more heavily on representative samples. We note, however, that this makes research on vegetarians and vegans a lot more difficult, since the representative sample we used included over 30,000 Germans, but only included 1,203 vegetarians and 128 vegans. One possibility to overcome this difficulty might be to convince other large panel-studies to include a question on diet, which we believe might not only improve psychological research into dietary behaviors, but would also be interesting for economic or medical research.

Taking a closer look at the results of our main study, as well as the prior evidence provided by Kessler, Holler [30] and Kováč and Halama [29], differences in personality between vegetarians and vegans seem to be either small and restricted to Openness or Neuroticism (or both), or are non-existent. To put our effect sizes of *d* = 0.18 (difference in Neuroticism in our main study) into perspective: When we randomly compared vegetarians and vegans in our main study, the vegetarians had a smaller Neuroticism value in only 48% of the cases. This idea is further supported by the pattern of the exploratory analyses we performed, where we found no group differences between vegetarians and vegans in measures regarding health status, political interest, or life satisfaction and affective wellbeing, when controlling for possibly confounding variables. While these differences we found are relatively small given Cohen’s [37] taxonomy (but see [38, 39], for what may be more realistic magnitudes of effect sizes in psychological research), this does not mean that these effect sizes should be neglected. Eating a vegetarian or vegan diet, as is the case for many of the phenomena researched in psychology, is likely affected by multiple factors. Factors such as mood, availability, norms, values, or culture might affect diet, and it is therefore unrealistic to assume large effect sizes for the association between personality and diet. However, as pointed out by Greenwald, Banaji [40] and Götz, Gosling [41], although small effects are likely the norm in psychological sciences, they can still have substantial consequences. The areas in which we argued that our results might be informative (i.e., differences in health outcomes) are ones in which small effects for a vegetarian or vegan diet compared to an omnivore diet are often reported [42], and considering even small personality differences might either help to explain some of these results, or improve their robustness.

### Limitations and future directions

The data presented here included individuals from a Western country where a vegetarian or vegan diet may have a different cultural meaning than in other countries, and vegetarians and vegans might therefore not be representative for vegetarians and vegans worldwide. However, this study is the first study on this topic to date that uses data from a large representative sample.

Our main study was slightly underpowered for the effect that we found. Given our sample sizes, the effect of *d* = 0.18 could be found with a power of 61%. We therefore cannot exclude the possibility that the effect for Neuroticism was a chance finding, and larger sample sizes are necessary. As previously noted, however, drawing a large enough representative samples that included an appropriate number of vegans might pose a challenge to researchers.

Research suggests that not only the food eaten might be a factor for differences between vegetarians and vegans, but the degree to which people identify with their eating behavior [43]. Future research into personality differences might consider including measures of vegetarian or vegan identity for more nuanced insights into differences between and within dietary groups.

Lastly, we believe that future research into differences in life outcomes (e.g., health, longevity, political engagement) between vegetarians and vegans that includes personality as a control variable might be interesting. This research could not only provide information on the reliability of prior findings, but also on the validity and importance of differences in personality between vegetarians and vegans.

## Conclusion

We provided mixed evidence on differences between vegetarians and vegans, and cannot conclude with certainty that there are consistent differences between vegetarians and vegans with regard to the Big Five. We believe, however, that this outcome supports the idea that there are either no personality differences between vegetarians and vegans, or that there are only small differences in Openness and/or Neuroticism. Further, we believe that our pattern of results is of value for research on eating behavior. We cautiously suggest that researchers who investigate differences between dietary groups and who combine vegetarians and vegans into a single group might conduct additional analyses with both groups separated (if sample sizes allow it), since we cannot exclude the possibility of systematic differences in personality between both groups. If replicated, these results might be of value for models and explanations about how and why vegetarians and vegans are different from each other. To reach such conclusions, however, more and better data are necessary, and we suggest researchers in the field to try to convince panel providers to include variables on vegetarian or vegan diet in their surveys.
